# Supplementary material for: A cell-type deconvolution meta-analysis of whole blood EWAS reveals lineage-specific smoking-associated DNA methylation changes
Source: Nat Commun. 2020 Sep 22;11:4779. doi: 10.1038/s41467-020-18618-y (PMC7508850; doi:10.1038/s41467-020-18618-y)
Supplement: Supplementary file 1 — Supplementary Information [file 41467_2020_18618_MOESM1_ESM.pdf]

Supplementary Information for "*A cell-type deconvolution meta-analysis of whole blood EWAS reveals lineage-specific smoking-associated DNA methylation changes*"

You et al

SUPPLEMENTARY FIGURES

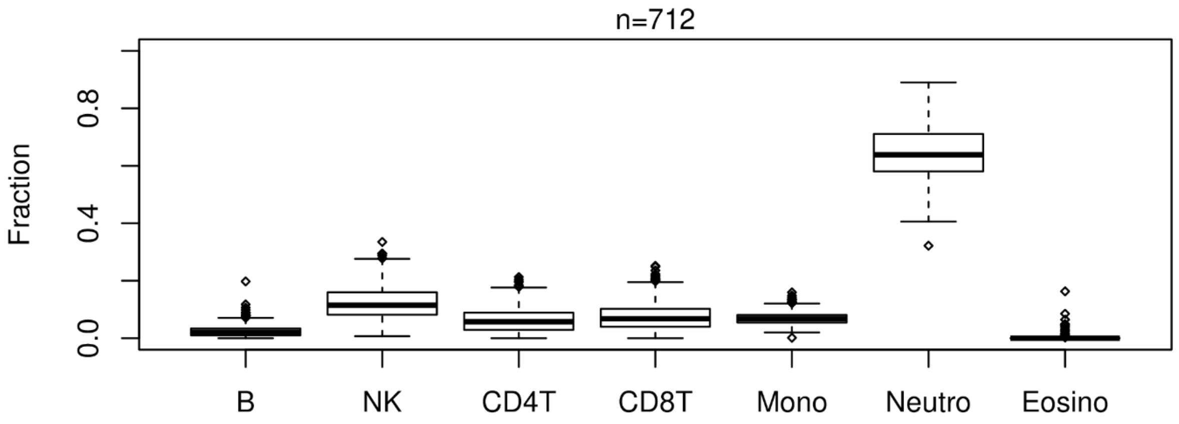

Supplementary Figure 1: Blood cell type fraction estimates derived from EpiDISH. Blood cell subtype fractions as estimated using the EpiDISH algorithm in the TZH cohort. Number of samples is indicated above plot. Horizontal line within boxes indicate median, box-boundaries the inter-quartile range, and whiskers extend to 1.5 times this range.

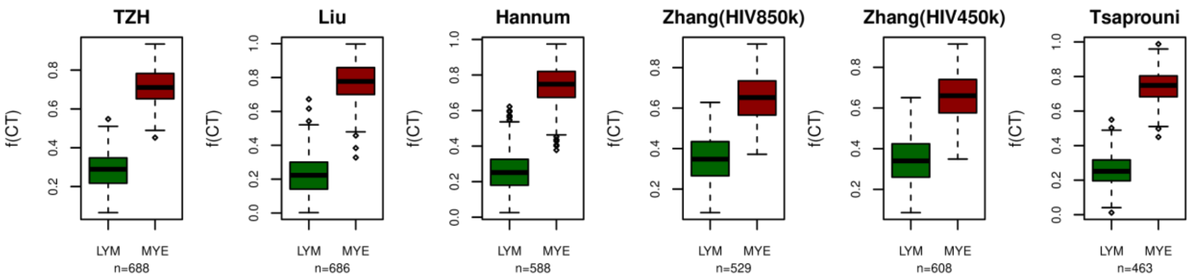

Supplementary Figure 2: Lymphoid and myeloid fractions across all 6 whole blood cohorts. For each of the 6 whole blood cohorts, we used EpiDISH to estimate cell-type fractions for each of the 7 main blood cell subtypes (CD4T, CD8T, NK, B, Mono, Neu, Eosin.), which we then summarize at the level of lymphoid and myeloid cells, as shown. Number of whole blood samples in each cohort is given. Horizontal line within boxes indicate median, box-boundaries the inter-quartile range, and whiskers extend to 1.5 times this range.

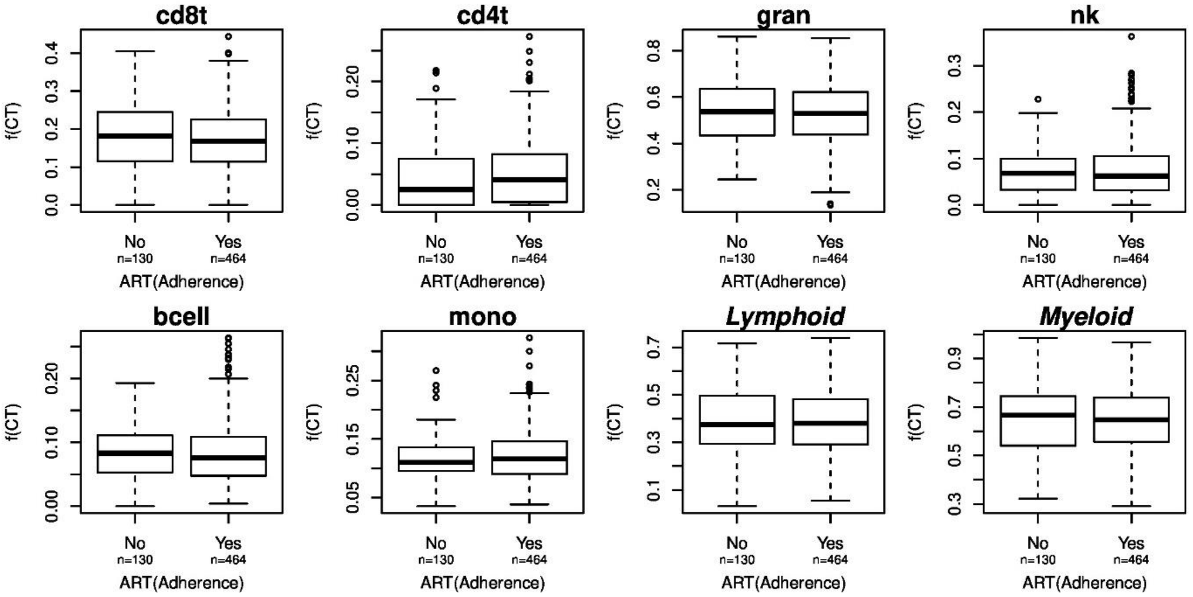

Supplementary Figure 3: Cell-type fractions vs ART in Zhang(HIV450k). For 6 main blood cell subtypes and the total lymphoid and myeloid fractions, we compare corresponding cell-type fractions (f(CT),y-axis) in the Zhang(HIV450k) cohort against adherence to anti-retroviral therapy (ART). Number of HIV patients complying with ART or not is given. In this figure, the granulocyte fraction is the total of neutrophil and eosinophil fraction. Horizontal line within boxes indicate median, box-boundaries the inter-quartile range, and whiskers extend to 1.5 times this range.

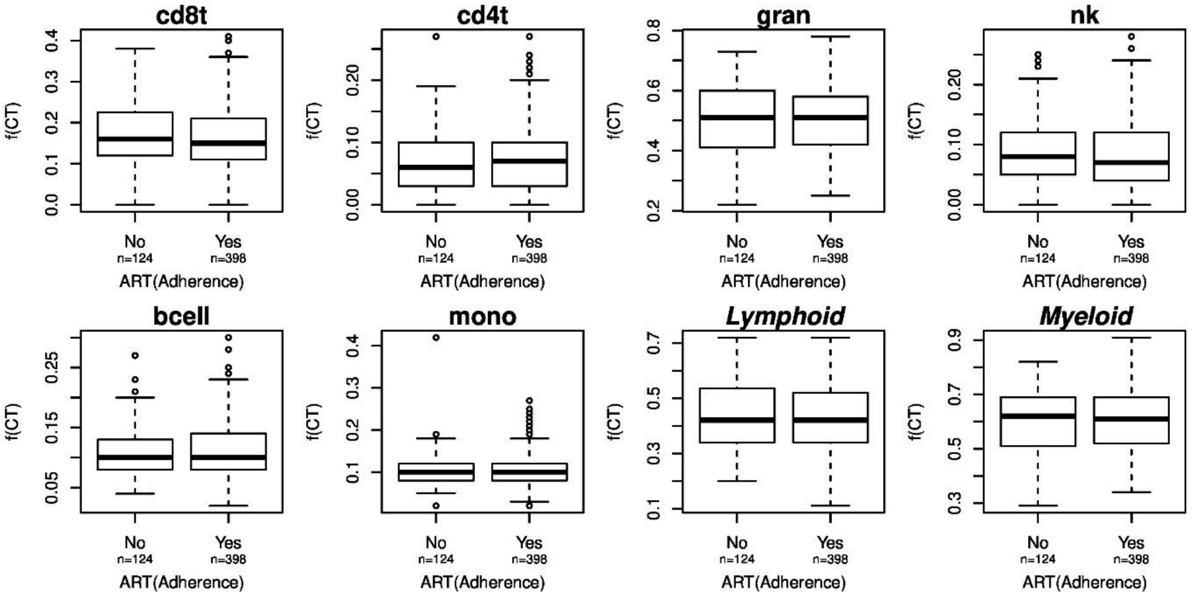

Supplementary Figure 4: Cell-type fractions vs ART in Zhang(HIV850k). For 6 main blood cell subtypes and the total lymphoid and myeloid fractions, we compare corresponding cell-type fractions (f(CT),y-axis) in the Zhang(HIV850k) cohort against adherence to anti-retroviral therapy (ART). Number of HIV patients complying with ART or not is given. Horizontal line within boxes indicate median, box-boundaries the inter-quartile range, and whiskers extend to 1.5 times this range.

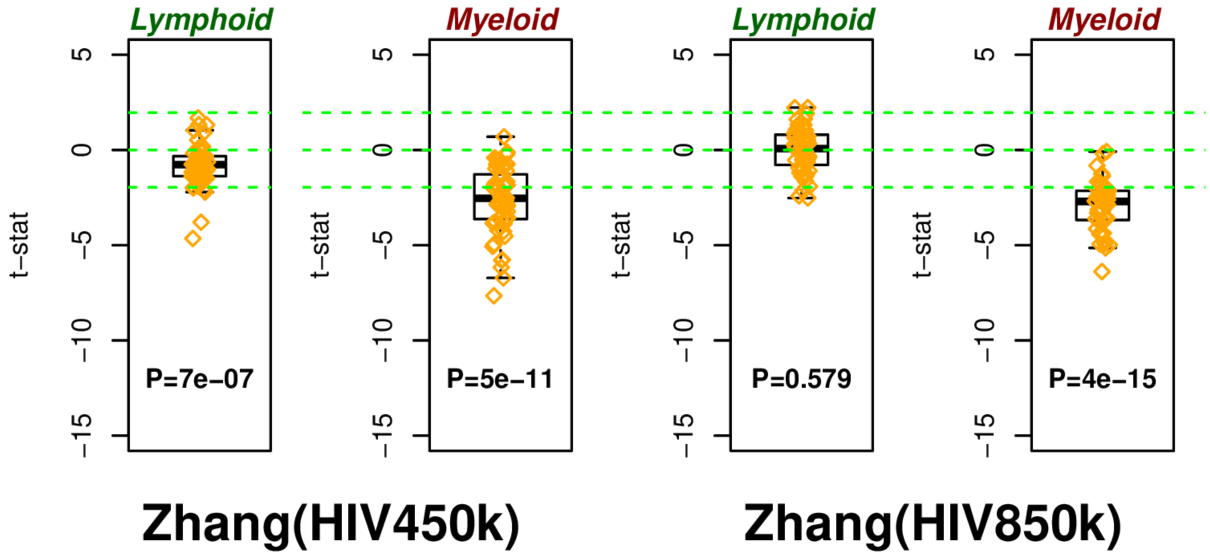

Supplementary Figure 5: Cell-type specific associations of gold-standard CpGs with smoking in HIV cohorts. Boxplots of t-statistics of association of DNAm with smoking in the whole blood HIV cohorts of Zhang et al, as derived with CellDMC for both lymphoid and myeloid lineages. Boxplots only display the 60 gold-standard smoking hypomethylated CpGs from Gao & Brenner. P-value derives

from a one-tailed Wilcoxon rank sum test. Horizontal line within boxes indicate median, box-boundaries the inter-quartile range, and whiskers extend to 1.5 times this range.

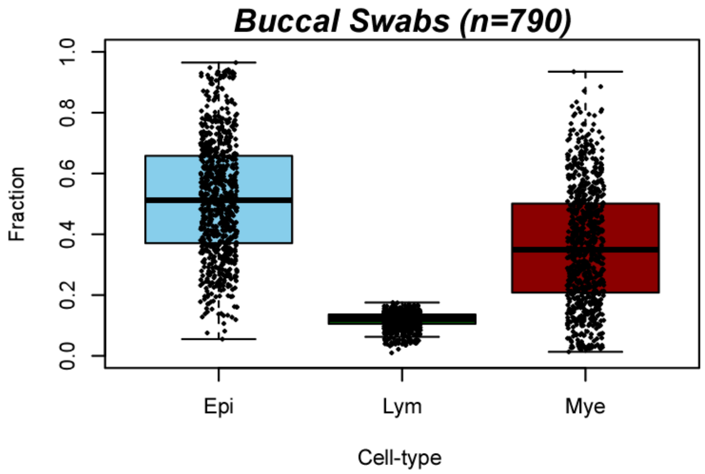

Supplementary Figure 6: Estimates of cell-type fractions in buccal swabs using HEpiDISH. Estimates of the fractions of total epithelial, total lymphoid and total myeloid cells in the 790 buccal swabs. Horizontal line within boxes indicate median, box-boundaries the inter-quartile range, and whiskers extend to 1.5 times this range.

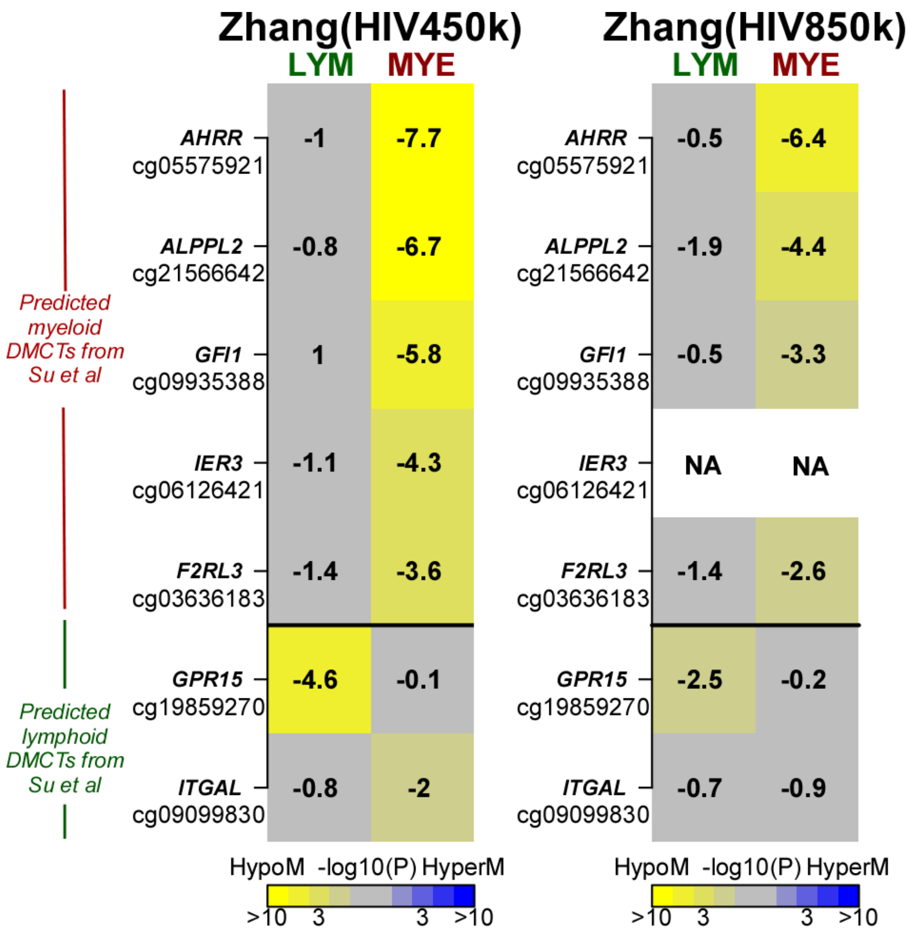

Supplementary Figure 7: CellDMC predictions in HIV cohorts. Heatmaps of myeloid and lymphoid significance P-values, as derived from CellDMC, in 2 separate HIV cohorts from Zhang et al, and for a panel of 7 CpGs which Su et al showed to exhibit myeloid and lymphoid specific hypomethylation in smokers. Significance of P-values is denoted by color, and the corresponding t-test statistic values are displayed in the heatmap. P-values derive from the t-test in the CellDMC model and are two-tailed.

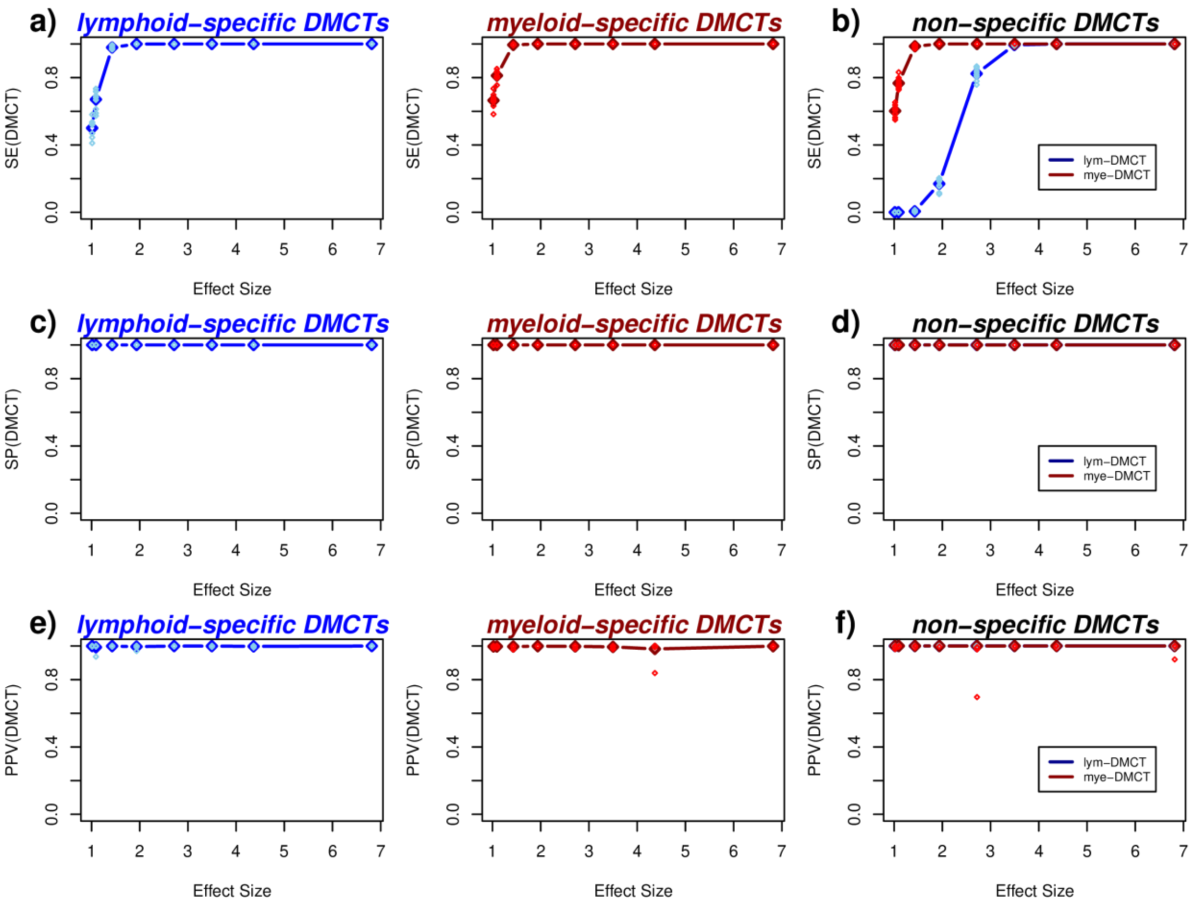

Supplementary Figure 8: Power estimates for simulation model using n=600. a) Plot of the sensitivity to detect lymphoid and myeloid specific DMCTs (y-axis) vs. the smoking effect size (x-axis). Data points represent the mean sensitivity, as obtained over 10 different Monte-Carlo simulations, encompassing 482077 CpGs of which 1000 are DMCTs. Datapoints are for n=600 (300 cases and controls). Each sample is an in-silico mixture of real DNAm profiles representing one purified CD4+ T-cell and one monocyte sample. For each case sample, 1000 DMCTs at the given average effect size in only one of the two cell-types was generated. The sensitivities for each of the 10 Monte-Carlo runs are shown in skyblue/red. **b)** As a), but now for the scenario where the DMCTs are introduced at the same CpG in both cell-types. Sensitivity to detect the DMCT in the lymphoid and myeloid lineage is shown. **c-d)** As a-b), but now for the specificity. **e-f)** As a-b), but now for the precision or positive predictive value (PPV).
