# Supplementary material for: A cell-type deconvolution meta-analysis of whole blood EWAS reveals lineage-specific smoking-associated DNA methylation changes
Source: Nat Commun. 2020 Sep 22;11:4779. doi: 10.1038/s41467-020-18618-y (PMC7508850; doi:10.1038/s41467-020-18618-y)
Supplement: Supplementary file 3 — Description of Additional Supplementary Files [file 41467_2020_18618_MOESM3_ESM.pdf]

Description of Additional Supplementary Files

File Name: Supplementary Data 1
Description: Participant characteristics of the 7 cohorts, including numbers of samples, male/sex ratio and age, stratified by smoking-status. For age, we provide the average age+/- standard deviation within each smoking-status stratum.

File Name: Supplementary Data 2
Description: Table of myeloid-DMCTs (FDR<0.3). Columns label the CpG identifier, the t-statistics from CellDMC for each of the 7 cohorts, Stouffer's z-statistic, P-value and two FDR estimates (Stouffer & Empirical).

File Name: Supplementary Data 3
Description: Table of lymphoid-DMCTs (FDR<0.3). Columns label the CpG identifier, the t-statistics from CellDMC for each of the 7 cohorts, Stouffer's z-statistic, P-value and two FDR estimates (Stouffer & Empirical).

File Name: Supplementary Data 4
Description: Table lists the number of overlapping CpGs of the myeloid and lymphoid DMCTs (FDR<0.3) found among the list of smoking-associated CpGs from Joehanes et al (FDR<0.05). Rows label the number of studies of our meta-analysis (maximum number of studies=7).

File Name: Supplementary Data 5
Description: Table listing the 108 hypomethylated myeloid DMCTs from our meta-analysis which map to a DNase Hypersensitive Site (DHS) (as defined by the eFORGE algorithm) in inflammatory macrophages. Table columns label Genome build, Chromosome, Position of CpG along chromosome, Gene Symbol and gene region.

File Name: Supplementary Data 6
Description: Table listing the 63 hypermethylated myeloid DMCTs from our meta-analysis which map to a DNase Hypersensitive Site (DHS) (as defined by the eFORGE algorithm) in acute myeloid leukemia. Table columns label Genome build, Chromosome, Position of CpG along chromosome, Gene Symbol and gene region.
