# Supplementary material for: A cell-type deconvolution meta-analysis of whole blood EWAS reveals lineage-specific smoking-associated DNA methylation changes
Source: Nat Commun. 2020 Sep 22;11:4779. doi: 10.1038/s41467-020-18618-y (PMC7508850; doi:10.1038/s41467-020-18618-y)
Supplement: Supplementary file 5 — Reporting summary [file 41467_2020_18618_MOESM5_ESM.pdf]

nature research

Corresponding author(s): Andrew E Teschendorff

Last updated by author(s): Aug 10, 2020

Reporting Summary

Nature Research wishes to improve the reproducibility of the work that we publish. This form provides structure for consistency and transparency in reporting. For further information on Nature Research policies, see Authors & Referees and the Editorial Policy Checklist .

Statistics

For all statistical analyses, confirm that the following items are present in the figure legend, table legend, main text, or Methods section.

n/a	Confirmed	
☐	☒	The exact sample size (*n*) for each experimental group/condition, given as a discrete number and unit of measurement
☐	☒	A statement on whether measurements were taken from distinct samples or whether the same sample was measured repeatedly
☐	☒	The statistical test(s) used AND whether they are one- or two-sided<br>*Only common tests should be described solely by name; describe more complex techniques in the Methods section.*
☐	☒	A description of all covariates tested
☐	☒	A description of any assumptions or corrections, such as tests of normality and adjustment for multiple comparisons
☐	☒	A full description of the statistical parameters including central tendency (e.g. means) or other basic estimates (e.g. regression coefficient) AND variation (e.g. standard deviation) or associated estimates of uncertainty (e.g. confidence intervals)
☐	☒	For null hypothesis testing, the test statistic (e.g. *F*, *t*, *r*) with confidence intervals, effect sizes, degrees of freedom and *P* value noted<br>*Give P values as exact values whenever suitable.*
☐	☒	For Bayesian analysis, information on the choice of priors and Markov chain Monte Carlo settings
☐	☒	For hierarchical and complex designs, identification of the appropriate level for tests and full reporting of outcomes
☐	☒	Estimates of effect sizes (e.g. Cohen's *d*, Pearson's *r*), indicating how they were calculated

Our web collection on statistics for biologists contains articles on many of the points above.

Software and code

Policy information about availability of computer code

Data collection	No particular software was used to collect data.
Data analysis	All statistical analyses were performed using R-version 3.6.2 freely available from cran.r-project.org<br>the following R/BioC packages were used: EpiDISH v2.0.2 , locfdr v1.1-8 , impute v1.58.0 , minfi v1.30.0 , sva_3.32.1, all freely available either from www.bioconductor.org or cran.r-project.org .<br>The code for the meta-analysis is part of the EpiDISH package, freely available from http://bioconductor.org/packages/devel/EpiDISH<br>eFORGE2 was run using the server at https://eforge.altiusinstitute.org/<br>BMIQ was run using R-code, freely available from https://aeteschendorff-lab.github.io/software/BMIQ

For manuscripts utilizing custom algorithms or software that are central to the research but not yet described in published literature, software must be made available to editors/reviewers. We strongly encourage code deposition in a community repository (e.g. GitHub). See the Nature Research guidelines for submitting code & software for further information.

Data

Policy information about availability of data

All manuscripts must include a data availability statement. This statement should provide the following information, where applicable:

- Accession codes, unique identifiers, or web links for publicly available datasets
- A list of figures that have associated raw data
- A description of any restrictions on data availability

All data analyzed in this manuscript are publicly available from GEO (http: www.ncbi.nlm.nih.gov/geo/) under accession numbers GSE42861 (Liu et al dataset), GSE40279 (Hannum et al dataset), GSE50660 (Tsaprouni et al dataset), GSE117859 & GSE117860 (Zhang et al dataset). The buccal swab DNAm data is only available

by submitting data requests to mrclha.swiftinfo@ucl.ac.uk; see full policy at http://www.nshd.mrc.ac.uk/data.aspx. Managed access is in place for this 73 year old study to ensure that use of the data are within the bounds of consent given previously by participants, and to safeguard any potential threat to anonymity since the participants are all born in the same week. The Illumina EPIC DNAm data for the TZH cohort can be viewed at NODE (https://www.biosino.org/node) under accession number OEP000260, or directly at https://www.biosino.org/node/project/detail/OEP000260 , and accessed by submitting a request for data-access.

Field-specific reporting

Please select the one below that is the best fit for your research. If you are not sure, read the appropriate sections before making your selection.

- [x] Life sciences
- [ ] Behavioural & social sciences
- [ ] Ecological, evolutionary & environmental sciences

For a reference copy of the document with all sections, see nature.com/documents/nr-reporting-summary-flat.pdf

Life sciences study design

All studies must disclose on these points even when the disclosure is negative.

Sample size	In our study, most of the data analysed is already in the public domain and therefore sample sizes were pre-determined. For the TZH cohort, we analysed over 700 samples, which is similar in size to some of the largest EWAS in blood performed to date. In our study, we have only included fairly large EWAS datasets, with the smallest study still containing over 450 samples. Power calculations that these sample sizes are adequate to identify blood cell-type specific differentially methylated cytosines was provided in our Zheng SC et al Nat Methods 2018 paper. Results contained in the current study support the view that we had adequate power. The meta-analysis performed over the 6-7 large EWAS sets further confirmed that we are adequately powered.
Data exclusions	In general, all samples and probes that passed our QC criteria, as detailed in the Methods section, were used.
Replication	In our study, we perform a meta-analysis over 6-7 large EWAS, precisely in order to assess reproducibility of our findings. Results obtained are highly consistent across all 7 studies, and also consistent with independent small-scale EWAS which assessed small panels of smoking-associated loci in purified blood cell subtype samples.
Randomization	For the TZH cohort, blood samples were randomized in relation to beadchipID, beadchip position, sample well, plate, year of sample collection, subcohort (Han vs Zhuang ethinicities), and all major epidemiological variables, including age, smoking, gender and BMI.
Blinding	This is not relevant to this study, as we perform a meta-analysis of smoking EWAS, where the phenotype (i.e .smoking status) needs to be known in advance in order to conduct the supervised analyses in each EWAS study.

Reporting for specific materials, systems and methods

We require information from authors about some types of materials, experimental systems and methods used in many studies. Here, indicate whether each material, system or method listed is relevant to your study. If you are not sure if a list item applies to your research, read the appropriate section before selecting a response.

Materials & experimental systems

n/a	Involved in the study	
x		Antibodies
x		Eukaryotic cell lines
x		Palaeontology
x		Animals and other organisms
	x	Human research participants
x		Clinical data

Methods

n/a	Involved in the study	
x		ChIP-seq
x		Flow cytometry
x		MRI-based neuroimaging

Human research participants

Policy information about studies involving human research participants

Population characteristics	This only applies to the TZH samples, since details of all other cohorts were provided in their respective publications. For the TZH cohort, we analysed 712 blood samples from Chinese individuals, of which 517 were Han Chinese and 189 Zhuang Chinese. For 6, no ethnic information was available. Mean Age was 55 years with a standard deviation of +/- 10 years, age-range was (19-71 years). The TZH cohort has a relatively high proportion of smokers and ex-smokers: 173 current smokers, 62 ex-smokers and 454 never-smokers. Importantly, for the TZH cohort, gender and smoking-status are strongly confounded: of the 173 current smokers, 170 were men and only 3 were women; of the 62 ex-smokers, 60 were men and 2 were women, and of the 454 never-smokers, 125 were men and 329 were women.
Recruitment	The TZH cohort study was conducted in the local communities in Zhengzhou, Taizhou and Nanning in China. Participants were recruited at a voluntary basis with the following criteria: 1) aged between 18 and 80; 2) with no self-reported major diseases; 3) both parents were locally born. We are not aware of any potential sources of self-selection bias other than that participants are likely to be more health-conscious than the average person. We don't think that this can influence the results presented in this manuscript. The Declaration of Helsinki Principles was followed and all participants provided written informed consent.
Ethics oversight	The TZH cohort study was conducted with the official approval of the Ethics Committee of the Shanghai Institutes for Biological Sciences (ER-SIBS-261410).

Note that full information on the approval of the study protocol must also be provided in the manuscript.
